# Extended harmonic mapping connects the equations in classical, statistical, fluid, quantum physics and general relativity

**DOI:** 10.1038/s41598-020-75211-5

**Published:** 2020-10-26

**Authors:** Xiaobo Zhai, Changyu Huang, Gang Ren

**Affiliations:** 1 The Molecular Foundry, Lawrence Berkeley National Laboratory, 1 Cyclotron Road, Berkeley, CA 94720 USA; 2 College of Science, Xi’an University of Science and Technology, Xi’an, 710054 Shaanxi China

**Keywords:** Statistical physics, thermodynamics and nonlinear dynamics, Theoretical physics, General relativity and gravity, Fluid dynamics

## Abstract

One potential pathway to find an ultimate rule governing our universe is to hunt for a connection among the fundamental equations in physics. Recently, Ren et al. reported that the harmonic maps with potential introduced by Duan, named extended harmonic mapping (EHM), connect the equations of general relativity, chaos and quantum mechanics via a universal geodesic equation. The equation, expressed as Euler–Lagrange equations on the Riemannian manifold, was obtained from the principle of least action. Here, we further demonstrate that more than ten fundamental equations, including that  of classical mechanics, fluid physics, statistical physics, astrophysics, quantum physics and general relativity, can be connected by the same universal geodesic equation. The connection sketches a family tree of the physics equations, and their intrinsic connections reflect an alternative ultimate rule of our universe, i.e.*,* the principle of least action on a Finsler manifold.

## Introduction

One of the major unsolved problems in physics is a single unified theory of everything^1^. Gauge field theory has been introduced based on the assumption that forces are described as fermion interactions mediated by gauge bosons^2^. Grand unification theory, a special version of quantum field theory, unified three of the four forces, i.e., weak, strong, and electromagnetic forces. The superstring theory^3^, as one of the candidates of the ultimate theory of the universe^4^, first unified the four fundamental forces of physics into a single fundamental force via particle interaction. The particles were treated as vibrating strings (or strands) that interacted via joining or disjoining^5,6^. String theory (including quantum gravity theories^7^) is still waiting for direct validation by experiments.

An alternative strategy to unify all forces is to treat the motion of an object as a description of its differentiable trajectory instead of the nondifferentiable and quantified interaction of particles considered in the above theories. Many physicists, including A. Einstein, have searched for a theory to unify all forces on curved space-time^8^. Harmonic mapping (HM) theory on the Riemannian manifold is one of the candidates solved many nonlinear differential equations in physics^9^, including the magnetic monopole and soliton problems, nonlinear σ model, Heisenberg theory of ferromagnetism, and equations similar to the Einstein gravity equation^10–13^. The axisymmetric gravitational equation in general relativity was also provided as a solution of the Ernst's equation^14^ by solving the geodesic equation^15^. The applications of HM have also been used to describe physical processes, such as grid generation in the field of computational fluid dynamics^16^, and ray tracing and seismic propagation^17^. Unfortunately, HM has not yet been show a solution of chaos or quantum mechanics equations.

Recently, Ren et al. reported that extended harmonic mapping (EHM) contains the solutions of Schrödinger equation and two classical chaotic equations^18,19^. EHM was first reported in 1991 by Duan in studying nonlinear differential equations^20^. In his EHM theory, a potential term was added to the geodesic equation in the HM. This additional term can be treated as a geodesic equation under a special force, which corresponds to a geodesic equation (without force) on a special Finsler surface. A similar theory, HM with potential, was also reported by Fardoun et al. independently in 1997^21^ and then developed by others^22–30^. Moreover, a different form of HM with potential was introduced in 2009 and then used by Niu et al*.* in studying the motion of light in heteronomous media, in which an equivalent to the geodesic in a 4-D Riemannian space was introduced with a potential term in the canonical equation^31^. Considering that the EH,a special case of the EHM, contains a solution of the Ernst formulation of Einstein's equations in general relativity^15,32–34^, the EHM sheds light on the unification of different actions in the least action principle (LAP).

Here, we further demonstrate that more than ten important equations of physics can be derived from the geodesic equation in EHM under specific defined metrics and potentials. The equations include the Chandrasekhar equation and the Lane-Emden equation in astrophysics, the electrostatic force equation, the equation of Newton's second law, the equation of Newton's law of universal gravitation, the equation of a one-dimensional (1-D) spring, 1-D damped vibration differential equation, the drag equation, and the equation of Stoke’s law in fluid physics. The connection among those questions via a universal geodesic equation in the EHM may provide a pathway to hunt the universal theory through their mathematical connections.

### The extended harmonic map (EHM)

The HM theory^10–12^, as a branch of mathematical physics, was developed decades ago^35^ and used to study the relationship between two general curved (pseudo-)Riemannian manifolds via “least expanding/curving” maps in the target space. Dirichlet energy, an action in HM, has been functionally used as a generalization of the original kinematic energy of classical mechanics^35^. In a special case, the Ernst formulation of Einstein's equations for axisymmetric situations in general relativity can be derived^15,32–34^. The theory of a unifying framework has also been used to describe σ models and Yang-Mills fields^36^.

In the HM, M and N are two Riemannian manifolds with local coordinates ${\mathrm{x}}^{\mu } (\mu =\mathrm{1,2},\dots ,m)$ on M and local coordinates ${\Phi }^{A} \left(\mathrm{A}=\mathrm{1,2},\dots ,\mathrm{n}\right)$ on N. The metrics on M and N are denoted by

1 $$\begin{gathered} dl^{2} = g_{\mu \nu } (x)dx^{\mu } dy^{\nu } ;\quad {\dim}\left( {\text{M}} \right) = {\text{m}} \hfill \\ dL^{2} = G_{AB} \left( {\Phi } \right)d{\Phi }^{A} d{\Phi }^{B} ;\quad {\dim}\left( {\text{N}} \right) = {\text{n}} \hfill \\ \end{gathered}$$

respectively. A mapping

$$\Phi : M \to N$$

2 $$x \to\Phi (x)$$

is called an HM^10–12^, when the action $I$ satisfies the Euler-Lagrange equation resulting from the variational principle (the principle of least action) $\delta I=0$, in which the action is defined as follows:

3 $$I=\int {d}^{n}x\sqrt{g}\left[-\frac{1}{2}{g}^{\mu \nu }{\partial }_{\mu }{\Phi }^{A}{\partial }_{\nu }{\Phi }^{B}{G}_{AB}\left(\Phi \right)\right]$$

where ${\partial }_{\mu }=\frac{\partial }{\partial {x}^{\mu }}$. The condition for a map to be harmonic is given by the Euler–Lagrange geodesic equation

4 $$\frac{\partial L}{\partial {\Phi }^{A}}-{\partial }_{\mu }\frac{\partial L}{\partial \left({\partial }_{\mu }{\Phi }^{A}\right)}=0; A=\mathrm{1,2},\dots ,n$$

where

5 $$L=-\frac{1}{2}{g}^{\mu \nu }{\partial }_{\mu }{\Phi }^{A}{\partial }_{\nu }{\Phi }^{B}{G}_{AB}\left(\Phi \right)\sqrt{g}$$

The geodesic Eq. (4) follows the notational convention of Wald’s equation^37^. By substituting Eq. (5) into Eq. (4), we can obtain the Euler–Lagrange equations (or geodesic equations) of the HM given by

6 $$\frac{1}{\sqrt{g}}{\partial }_{\mu }\left({\sqrt{g}{g}^{\mu \nu }{\partial }_{\nu }\Phi }^{A}\right)+{\Gamma }_{BC}^{A}{\partial }_{\mu }{\Phi }^{B}{\partial }_{\nu }{\Phi }^{C}{g}^{\mu \nu }=0,$$

where ${\Gamma }_{BC}^{A}$ are the Christoffel symbols on manifold N given by

7 $${\Gamma }_{BC}^{A}=\frac{1}{2}{G}^{AD}\left[\frac{\partial {G}_{BD}}{{\partial\Phi }^{C}}+\frac{\partial {G}_{CD}}{{\partial\Phi }^{B}}-\frac{\partial {G}_{BC}}{{\partial\Phi }^{D}}\right].$$

In the 2-D case, the above Euler–Lagrange Eq. (6) provided a solution of the Ernst equation in Einstein’s general relativity^13–15^.

The EHM is extended from HM by added a potential energy term to the action $I$ in Eq. (3), since the traditional HM contains only kinetic energy term^18,20^. Thus, the action in the EHM is given by

8 $$I=\int {d}^{n}x\sqrt{g}[-\frac{1}{2}{g}^{\mu \nu }{\partial }_{\mu }{\Phi }^{A}{\partial }_{\nu }{\Phi }^{B}{G}_{AB}\left(\Phi \right)+V(\Phi )].$$

**Figure 1:**
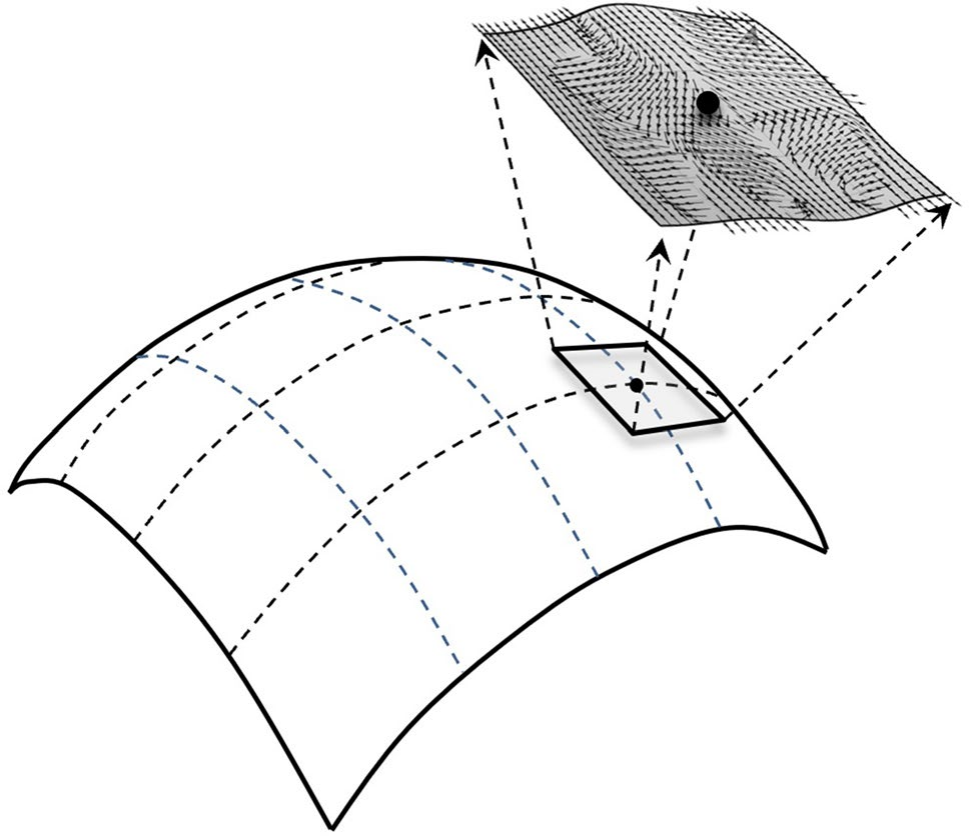
The schematic of the extended harmonic map (EHM) theory. The EHM can be understood as a local potential field added to the conventional harmonic map (HM). This addition changes the local space curvature and generates anisotropic/asymmetric space-time, which can be described as a special case of Finsler surface.

The additional item, $V\left(\Phi \right)=V\left({\Phi }^{1},{\Phi }^{1},{\dots .,\Phi }^{n}\right)$, is the potential function of ${\Phi }^{A}$^20^. This energy term changes the trajectory of the object motion, which corresponds to a higher-dimensional anisotropic/asymmetric space, as a special case of the Finsler manifold (Fig. 1). When $V\left(\Phi \right)=0$, the EHM turns into the traditional HM. The Euler-Lagrange’s equations of the EHM were initially reported by Duan in 1991^20^ for studying the traveling wave solutions of nonlinear partial differential equations^20^. A similar concept of a HM with potential was also introduced independently by Fardoun and Ratoo lately ^21^. The development of the HM with potential has been conducted by many others^25,29,38–40^.

In the EHM, the Euler–Lagrange equations, or the geodesic Eq. (4), become

9 $$\frac{1}{\sqrt{g}}{\partial }_{\mu }\left({\sqrt{g}{g}^{\mu \nu }{\partial }_{\nu }\Phi }^{A}\right)+{\Gamma }_{BC}^{A}{\partial }_{\mu }{\Phi }^{B}{\partial }_{\nu }{\Phi }^{C}{g}^{\mu \nu }+{G}^{AB}\frac{\partial V(\Phi )}{{\partial\Phi }^{B}}=0,$$

when

10 $$L=-\frac{1}{2}{g}^{\mu \nu }{\partial }_{\mu }{\Phi }^{A}{\partial }_{\nu }{\Phi }^{B}{G}_{AB}\left(\Phi \right)\sqrt{g}+V(\Phi )\sqrt{g}.$$

In the case of that (i) ${\Phi }^{A} \left(\mathrm{A}=\mathrm{1,2},\dots ,\mathrm{n}\right)$ are functions solely of the argument *σ* and *σ* is a function of $x^{\mu }$ on the manifold M, (ii) given the function $\sigma =\sigma (x)$ (which was also called the harmonic coordinate) satisfying the Laplace-Beltrami equations, $\frac{1}{\sqrt{g}}{\partial }_{\mu }\left(\sqrt{g}{g}^{\mu \nu }{\partial }_{\nu }\sigma \right)=0$, the Euler-Lagrange Eq. (9) can be written as the following geodesic equation, which was originally shown in Duan’s paper (as Eq. (7))^21^, i.e.*,*

11 $${\frac{{d}^{2}{\Phi }^{A}}{d{\sigma }^{2}}+{\Gamma }_{BC}^{A}\frac{d{\Phi }^{B}}{d\sigma }\frac{d{\Phi }^{C}}{d\sigma}}=-G^{AB}\frac{\partial U(\Phi )}{{\partial\Phi }^{B}},$$

where $U\left(\Phi \right)=\frac{V(\Phi )}{{g}^{\mu \nu }{\partial }_{\mu }\sigma {\partial }_{\nu }\sigma }$ is a potential function of ${\Phi }^{A}$, and ${\Gamma }_{BC}^{A}$ are the Christoffel symbols on manifold N given by Eq. (7).

The equation (11) can be understood as the geodesic equation of a particle on Riemannian manifold N subject to an external force ${F}^{A}{=-G}^{AB}\frac{\partial U(\Phi )}{{\partial\Phi }^{B}}$. When the external force vanishes, i.e., ${F}^{A}=0 (A=\mathrm{1,2},\dots , n)$, the Eq. (11) becomes equivalent to the traditional geodesic equations on manifold N. The Ernst equation in Einstein’s general relativity can be obtained from the traditional geodesic equations in a specific 2-D space-time^13,15^.

Equation (11) is a physical dynamical equation; thus, EHM can be considered as a dynamic form of the tranditional HM, which has a similar situation to that the general relativity is the dynamic version of the special relativity, and the Newton’s second law is a dynamic version of Newton’s first law. In the EHM, the isotropy and symmetry of Riemann geometry is interrupted by the added potential term, the geodesic equation under potential on a Riemannian manifold could be considered as a standard geodesic equation (without force) on a special Finsler manifold.

### In the case of a 2-dimensional manifold

For a simple case, M is the pseudo-Euclidean space-time, N is a 2-D manifold with coordinates $\left\{{\Phi }^{1},{\Phi }^{2}\right\}$, and ${\Phi }^{1}$ and ${\Phi }^{2}$ are functions of an argument $\sigma =\sigma \left(x\right)$, where ${\Phi }^{2}=\sigma$, i.e.,

12 $${\Phi }^{1}=\Phi \left(\sigma \right); {\Phi }^{2}=\sigma .$$

The Euler-Lagrange equations (the geodesic Eq. (11)) can be rewritten as follows^18,19^

13 $$\frac{{d}^{2}\Phi }{d{\sigma }^{2}}+2\left({\Gamma }_{12}^{1}-\frac{{\Gamma }_{11}^{1}{\Gamma }_{12}^{2}}{{\Gamma }_{11}^{2}}\right)\frac{d\Phi }{d\sigma }+\left({\Gamma }_{22}^{1}-\frac{{\Gamma }_{11}^{1}{\Gamma }_{22}^{2}}{{\Gamma }_{11}^{2}}\right)+\left[{G}^{11}\frac{\partial U\left(\Phi ,\sigma \right)}{\partial\Phi }+{G}^{12}\frac{\partial U\left(\Phi ,\sigma \right)}{\partial \sigma }-\frac{{\Gamma }_{11}^{1}}{{\Gamma }_{11}^{2}}\left({G}^{21}\frac{\partial U\left(\Phi ,\sigma \right)}{\partial\Phi }+{G}^{22}\frac{\partial U\left(\Phi ,\sigma \right)}{\partial \sigma }\right)\right]=0.$$

### Under a specific diagonal metric

Given that the metrics on manifold N are diagonal:

14 $${G}_{11}={e}^{\Phi +\sigma }; {G}_{12}=0; {G}_{21}=0; {G}_{22}=\left(R-1\right){e}^{\Phi +\sigma },$$

i.e.

14’ $${G}^{11}={e}^{-(\Phi +\sigma )}; {G}^{12}=0; {G}^{21}=0; {G}^{22}=\frac{1}{R-1}{e}^{-(\Phi +\sigma )},$$

where $R$ is named as a “reflecting/projecting angle” that can be a constant or a variable. Christoffel’s symbols (Eq. 7) on the 2-D manifold is calculated as follows:

15 $${\Gamma }_{11}^{1}=\frac{1}{2}; {\Gamma }_{12}^{1}=\frac{1}{2}; {\Gamma }_{22}^{1}=-\frac{1}{2}\left(R-1\right); {\Gamma }_{11}^{2}=-\frac{1}{2\left(R-1\right)}; {\Gamma }_{12}^{2}=\frac{1}{2}; {\Gamma }_{22}^{2}=\frac{1}{2}\left(1+\frac{{\partial }_{\sigma }R}{R-1}\right).$$

By substituting Eqs. (14) and (15) into Eq. (13), the geodesic Eq. (13) can be simplified as follows:

16 $$\frac{{d}^{2}\Phi }{d{\sigma }^{2}}+R\frac{d\Phi }{d\sigma }+\frac{{\partial }_{\sigma }R}{2}+({e}^{-\Phi -\sigma })\left[\frac{\partial U\left(\Phi ,\sigma \right)}{\partial\Phi }+\frac{\partial U\left(\Phi ,\sigma \right)}{\partial \sigma }\right]=0.$$

Equation (16) is a special geodesic equation in a 2-D manifold. This equation will be frequently used to derive the important equations in physics under a specific given reflection angle $R$ and potential $U\left(\Phi ,\sigma \right)$ in the following sections.

### The solutions of the equations in quantum physics

Recently, Ren, et al*.* reported the geodesic Eq. (16) contains a solution of the Schrödinger equation (for a 1-D harmonic oscillator) and two solutions of the classical chaos equations (the nonlinear equation for a harmonic system in periodic fields and the equation for a parametrically excited pendulum)^18^. In brief, under a given reflecting/projecting angle $R$ and a potential field $U\left(\varphi ,\sigma \right)$,

17 $$R=0,$$

18 $$U\left(\varphi ,\sigma \right)={e}^{\varphi +\sigma } \left[-\frac{m}{2{\hbar }^{2}}K\varphi {\sigma }^{2}+\frac{m}{4{\hbar }^{2}}{K\sigma }^{2}+\frac{m}{2{\hbar }^{2}}K\varphi \sigma +\frac{m}{2{\hbar }^{2}}\left(2E-\frac{1}{2}K\right)\varphi -\frac{m}{2{\hbar }^{2}}K\sigma +\frac{m}{2{\hbar }^{2}}\left(\frac{3}{4}K-E\right)\right],$$

where $E$, $K$, $m$, $\hbar$ are the constants, the geodesic Eq. (16) turns into

19 $$\frac{{d}^{2}\varphi (\sigma )}{d{\sigma }^{2}}=-\frac{2m}{{\hbar }^{2}}\left(E-\frac{1}{2}K{\sigma }^{2}\right)\varphi \left(\sigma \right).$$

This special form of geodesic Eq. (19) has the same form as that of the Schrödinger equation for a 1-D harmonic oscillator, i.e., $\frac{{d}^{2}\Psi (x)}{d{x}^{2}}=-\frac{2m}{{\hbar }^{2}}\left(E-\frac{1}{2}K{x}^{2}\right)\Psi \left(x\right)$, where $\Psi \left(x\right)$ (or $\varphi (\sigma )$) is the wave function (related to the probability of finding the particle), $E$ is called the eigenenergy (independent of $x$ or $\sigma$) in quantum mechanics (constant), and $K$ is the force constant (the force on the mass being $F=-Kx$, proportional to the displacement $x$ (or $\sigma$) towards the origin). In this case, the trajectory of a particle (the geodesic equation) in the 2-D manifold (formed by the wave function $\Psi \left(x\right)$ (or $\varphi (\sigma )$) and displacement $x$ (or $\sigma$)) follows the LAP.

### The solutions of four equations of classical mechanics

In this paper, we report that four classical mechanics equations can be derived from the geodesic Eq. (16) under specific given reflecting angles and potentials. For the first examples, when the reflecting/projecting angle $R=0$ and the potential function $U(\varphi ,\sigma )$ is defined as,

20 $$U(\varphi ,\sigma )={e}^{\varphi +\sigma }\left[-\frac{F}{2m}\right],$$

where $m$ and $F$ are constants, the geodesic Eq. (16) becomes

21 $$m\frac{{d}^{2}\varphi }{d{\sigma }^{2}}=F.$$

The Eq. (21) has a same formulation to the Newton’s second law, $F=m\frac{{d}^{2}x}{d{t}^{2}}$. Therefore, Thus the newton's second law can be discripted as the geodesic equation of a particle that travels on a 2-D manifold formed by displacement $x$ (or $\varphi$) and time $t$ (or $\sigma$) follows the LAP.

In the second example, given a potential function $U(\varphi ,\sigma )$

22 $$U(\varphi ,\sigma )={e}^{\varphi +\sigma }\left\{\frac{e{E}_{0}}{m(4+{\omega }^{2})}\left[2\mathrm{cos}\left(\omega \sigma +\theta \right)+\omega \mathrm{sin}\left(\omega \sigma +\theta \right)\right]\right\},$$

the geodesic Eq. (16) becomes the following equation:

23 $$m\frac{{d}^{2}\varphi }{d{\sigma }^{2}}=-e{E}_{0}\mathrm{cos}\left(\omega \sigma +\theta \right).$$

The Eq. (23) has a same form for an electron’s motion under an oscillating electric field, $m\frac{{d}^{2}x}{d{t}^{2}}=-e{E}_{0}\mathrm{cos}\left(\omega t+\theta \right)$. In an oscillating electric field along the x-axis, the electric field intensity is ${E}_{0}\mathrm{cos}\left(\omega t+\theta \right)$, where ${E}_{0}$ is the maximal electric field intensity and $\omega$ and $\theta$ are the frequency and phase of oscillation, respectively. The motion equation can be translated to be a trajectory (geodesic equation) of a particle that travels on a 2-D manifold formed by the displacement $x$ (or $\varphi$) and time $t$ (or $\sigma$) under the LAP.

In the third example, given a potential function $U(\varphi ,\sigma )$,

24 $$U(\varphi ,\sigma )={-e}^{\varphi +\sigma }\left[\frac{k}{4m}-\frac{k}{2m}\varphi \right],$$

the geodesic Eq. (16) becomes

25 $$m\frac{{d}^{2}\varphi }{d{\sigma }^{2}}=-k\varphi .$$

The Eq. (25) has a same form as the equation for a 1-D spring, where $k$ is the elasticity modulus, $m\frac{{d}^{2}x}{d{t}^{2}}=-kx$, suggesting the trajectory of the particle follows the PLA in the 2-D manifold formed by the displacement $x$ (or $\varphi$) and time $t$ (or $\sigma$).

In the fourth example, we define the potential function $U\left(\varphi ,\sigma \right),$

26 $$U(\varphi ,\sigma )={e}^{\varphi +\sigma }\left[\frac{k}{m\varphi }-\frac{2k}{m}{e}^{-2\varphi }\mathrm{Ei}(2\varphi )\right],$$

where $\mathrm{Ei}\left(2\sigma \right)=-{\int }_{-2\sigma }^{\infty }\frac{{e}^{-t}}{t}dt$ is the exponential integral function $\mathrm{Ei}\left(x\right)=-{\int }_{-x}^{\infty }\frac{{e}^{-t}}{t}dt$^42^. $\mathrm{Ei}\left(x\right)$ is not an elementary function in the Risch algorithm^42^, and $t$ is a meaningless variant for generating the integral function. The Eq. (16) becomes

27 $$m\frac{{d}^{2}\varphi }{d{\sigma }^{2}}=\frac{k}{{\varphi }^{2}}.$$

The Eq. (27) has the same formula as that of the electrostatic force equation between two charges ($\mathrm{q}$ and $\mathrm{Q}$): $F(x)=m\frac{{d}^{2}r}{d{t}^{2}}=\frac{1}{4\uppi {\varepsilon }_{0}}\frac{\mathrm{qQ}}{{r}^{2}}=\frac{k}{{r}^{2}}$, where $k=\frac{\mathrm{qQ}}{4\uppi {\varepsilon }_{0}}$ (${\varepsilon }_{0}$ is the vacuum permittivity or permittivity of free space and $r$ is the distance between the two charges). The result suggests that a particle travelling under electronic field  follows the geodesic equation in distance-time space under the PLA. Moreover, Eq. (27) also has the same formula as that of Newton's law of universal gravitation (between two masses, $\mathrm{m}$ and $\mathrm{M}$): $F\left(x\right)=m\frac{{d}^{2}r}{d{t}^{2}}=\mathrm{G}\frac{\mathrm{mM}}{{r}^{2}}=\frac{k}{{r}^{2}}$, where $k=\mathrm{GmM}$ ($\mathrm{G}$ is the gravitational constant and $r$ is the distance between the two charges). Thus, the trajectory of the particle travelling under gravity also follows the PLA in distance-time space.

The above examples can be understood as Euclidean geometric trajectories (descripted as a “projection”) of the Euler-Lagrange equations or geodesic equations on a Riemannian surface under specific manifold metrics (defined by a projecting angle) and potential fields (as “local environments under a specific super-force”).

### The solutions of two chaotic equations

Previously, we have reported that complex equations of classical mechanics, chaos equations, can be derived from geodesic Eq. (16)^18,19^. For a brief review, under a specific given reflecting angle $R=k\ne 0$, a non-zero constant, and potential field^18,19^

28 $$U\left(\varphi ,\sigma \right)={e}^{\varphi +\sigma }\left [\frac{1}{2}\alpha {\varphi }^{3}-\frac{3}{4}\alpha {\varphi }^{2}-\frac{1}{4}\left(2\beta -3\alpha \right)\varphi +\frac{1}{8}\left(2\beta -3\alpha \right)-\frac{2b}{{\omega }^{2}+4}\mathrm{cos}\left(\omega \sigma \right)-\frac{\omega b}{{\omega }^{2}+4}\mathrm{sin}\left(\omega \sigma \right)\right],$$

where k, α and β are control parameters, the Eq. (16) becomes

29 $$\frac{{d}^{2}\varphi }{d{\sigma }^{2}}+k\frac{d\varphi }{d\sigma }-\beta \varphi +\alpha {\varphi }^{3}=bcos\left(\omega \sigma \right).$$

The Eq. (29) has a same formula as the nonlinear chaotic equation for a harmonic system in a periodic field^43^, given by $\frac{{d}^{2}x}{d{t}^{2}}+k\frac{dx}{dt}-\beta x+\alpha {x}^{3}=bcos\left(\omega t\right)$. Thus, the trajectory of a particle follows the LAP in displacement ($\varphi$ or $x$) and time ($\sigma$ or $t$) space.

However, under the same reflecting angle $R=k\ne 0$ and a different potential function^18,19^

30 $$U\left(\varphi ,\sigma \right)={e}^{\varphi +\sigma }\left \{\frac{2}{5}\alpha \mathrm{sin}\left(\varphi \right)-\frac{1}{5}\alpha \mathrm{cos}\left(\varphi \right)+\frac{\beta }{25+6{\omega }^{2}+{\omega }^{4}}\left[\omega \left({\omega }^{2}+3\right)\mathrm{sin}\left(\omega \sigma \right)\mathrm{sin}\left(\varphi \right)-4\omega \mathrm{sin}\left(\omega \sigma \right)\mathrm{cos}\left(\varphi \right)+2\left({\omega }^{2}+5\right)\mathrm{cos}\left(\omega \sigma \right)\mathrm{sin}\left(\varphi \right)+\left({\omega }^{2}-5\right)\mathrm{cos}\left(\omega \sigma \right)\mathrm{cos}\left(\varphi \right)\right]\right\},$$

in which α and β are constants, the Eq. (16) becomes

31 $$\frac{{d}^{2}\varphi }{d{\sigma }^{2}}+k\frac{d\varphi }{d\sigma }+[\alpha +\beta \mathrm{cos}\left(\omega \sigma \right)]\mathrm{sin}\left(\varphi \right)=0.$$

This specific form of the geodesic equation is the same as the chaotic equation for a parametrically excited pendulum^44^, $\frac{{d}^{2}x}{d{t}^{2}}+k\frac{dx}{dt}+[\alpha +\beta \mathrm{cos}\left(\omega t\right)]\mathrm{sin}\left(x\right)=0$, where *k, α* and *β* are control parameters. In this case, the trajectory (geodesic equation) of a particle in the 2-D manifold formed by displacement ($\varphi$ or $x$) and time ($\sigma$ or $t$) also follows the LAP, although the displacement appears to be randomly distributed against time in forming a chaos phenomenon.

### The solutions of three statistical physics-related equations in astrophysics

In this paper, we report that three statistical physics equations can also be described as the solutions of the geodesic Eq. (16) under a specific projecting/reflecting angle and potential. Under a given variable reflecting angle of

32 $$R=\frac{2}{\sigma },$$

the Christoffel symbols become

33 $${\Gamma }_{11}^{1}=\frac{1}{2}; {\Gamma }_{12}^{1}=\frac{1}{2}; {\Gamma }_{22}^{1}=\frac{\sigma -2}{2\sigma }; {\Gamma }_{11}^{2}=\frac{\sigma }{2(\sigma -2)}; {\Gamma }_{12}^{2}=\frac{1}{2}; {\Gamma }_{22}^{2}=\frac{{\sigma }^{2}-2\sigma +2}{2\sigma (\sigma -2)}.$$

The Eq. (16) can be rewritten as follows:

34 $$\frac{{d}^{2}\varphi }{d{\sigma }^{2}}+\frac{2}{\sigma }\frac{d\varphi }{d\sigma }-\frac{1}{{\sigma }^{2}}+({e}^{-\mathrm{\varphi }-\sigma })\left[\frac{\partial U\left(\varphi ,\sigma \right)}{\partial \varphi }+\frac{\partial U\left(\varphi ,\sigma \right)}{\partial \sigma }\right]=0.$$

In the first example, when the potential function $U\left(\varphi ,\sigma \right)$ is given by

35 $$U(\varphi ,\sigma )={e}^{\varphi +\sigma }[-{e}^{-\varphi }-\frac{1}{\sigma }+2{e}^{-2\sigma }\mathrm{Ei}(2\sigma )],$$

where $\mathrm{Ei}\left(2\sigma \right)=-{\int }_{-2\sigma }^{\infty }\frac{{e}^{-t}}{t}dt$ is the exponential integral function $\mathrm{Ei}\left(x\right)=-{\int }_{-x}^{\infty }\frac{{e}^{-t}}{t}dt,$ ^41^ the geodesic Eq. (34) becomes

36 $$\frac{{d}^{2}\varphi }{d{\sigma }^{2}}+\frac{2}{\sigma }\frac{d\varphi }{d\sigma }-{e}^{-\varphi }=0.$$

Considering

37 $$\frac{{d}^{2}\varphi }{d{\sigma }^{2}}+\frac{2}{\sigma }\frac{d\varphi }{d\sigma }=\frac{1}{{\sigma }^{2}}\left({\sigma }^{2}\frac{{d}^{2}\varphi }{d{\sigma }^{2}}+2\sigma \frac{d\varphi }{d\sigma }\right)=\frac{1}{{\sigma }^{2}}\left[{\sigma }^{2}\frac{d}{d\sigma }\left(\frac{d\varphi }{d\sigma }\right)+\frac{d\left({\sigma }^{2}\right)}{d\sigma }\frac{d\varphi }{d\sigma }\right]=\frac{1}{{\sigma }^{2}}\frac{d}{d\sigma }\left({\sigma }^{2}\frac{d\varphi }{d\sigma }\right).$$

The Eq. (36) can be rewritten as

38 $$\frac{1}{{\sigma }^{2}}\frac{d}{d\sigma }\left({\sigma }^{2}\frac{d\varphi }{d\sigma }\right)={e}^{-\varphi }.$$

The Eq. (38) has the same form as the Emden-Chandrasekhar equation in astrophysics^45,46^, which is used to calculate physical variables, such as the density distribution near the center of a star, which is isothermal and satisfies the state equation $P=\rho \frac{{k}_{B}}{WH}T+\frac{4{\sigma }_{0}}{3c}{T}^{4}$. In the Emden-Chandrasekhar equation, $\varphi$ and $\sigma$ are related to the density $\rho$ and radius $r$ of the star, respectively, i.e.*,* $\varphi \text{=}\mathrm{ln}\frac{{\rho }_{C}}{\rho }, \sigma =r{\left(\frac{4\pi G{\rho }_{C}WH}{{k}_{B}T}\right)}^{1/2}$, where ${\rho }_{C}$ is the central density. The constant *G* is the gravitational constant, *W* is the mean molecular weight, *H* is the mass of a proton, *T* is the temperature of the star, ${k}_{B}$ is the Boltzmann constant, ${\sigma }_{0}$ is the Stefan–Boltzmann constant, and *c* is the speed of light. The result suggests that the trajectory (geodesic equation) of a particle in the 2-D manifold (formed by the density and radius) follows the LAP, although the equation was originally obtained from the Poisson equation of an isothermal gas sphere subjected to its own gravitational force.

In the second example, when the given potential energy function $U\left(\varphi ,\sigma \right)$ is

39 $$U(\varphi ,\sigma )={e}^{\varphi +\sigma }\left[-\frac{1}{\sigma }+2{e}^{-2\sigma }\mathrm{Ei}\left(2\sigma \right)+\frac{1}{2}{{\left({\varphi }^{2}-C\right)}^{3/2}-\frac{3}{2}e}^{-2\varphi }\Omega \left(\varphi \right)\right],$$

where $\mathrm{Ei}\left(2\sigma \right)=-{\int }_{-2\sigma }^{\infty }\frac{{e}^{-t}}{t}dt$ is the exponential integral function defined as $\mathrm{Ei}\left(x\right)=-{\int }_{-x}^{\infty }\frac{{e}^{-t}}{t}dt$^42^ and $\Omega \left(\varphi \right)=\int \varphi \sqrt{{\varphi }^{2}-C}{e}^{2\varphi }d\varphi$, the geodesic Eq. (34) becomes

40 $$\frac{{d}^{2}\varphi }{d{\sigma }^{2}}+\frac{2}{\sigma }\frac{d\varphi }{d\sigma }+{\left({\varphi }^{2}-C\right)}^{3/2}=0.$$

Considering the Eq. (37), the Eq. (40) can be rewritten as

41 $$\frac{1}{{\sigma }^{2}}\frac{d}{d\sigma }\left({\sigma }^{2}\frac{d\varphi }{d\sigma }\right)+{\left({\varphi }^{2}-C\right)}^{3/2}=0.$$

The Eq. (41) has the same form as the Chandrasekhar's white dwarf equation in astrophysics^46,47^, in which $\varphi$ is related to the density of a white dwarf (with boundary conditions $\varphi \left(0\right)=0$ and $\varphi {^{\prime}}\left(0\right)=0$) and $\sigma$ is the radius of the white dwarf. Although the Eq. (41) was originally developed based on the equilibrium statistical mechanics for self-gravitating systems in astrophysics, the distribution of the density against the radius follows the LAP.

In the third example, when the potential energy function $U\left(\varphi ,\sigma \right)$ is given by

42 $$U(\varphi ,\sigma )={e}^{\varphi +\sigma }[-\frac{1}{\sigma }+2{e}^{-2\sigma }\mathrm{Ei}\left(2\sigma \right)-{\left(-2\right)}^{-1-n}{e}^{-2\varphi }\Gamma (1+n,-2\varphi )],$$

where $\mathrm{Ei}\left(2\sigma \right)=-{\int }_{-2\sigma }^{\infty }\frac{{e}^{-t}}{t}dt$ is the exponential integral function $\mathrm{E}\mathrm{i}\left(x\right)=-{\int }_{-x}^{\infty }\frac{{e}^{-t}}{t}dt$^42^ and $\Gamma \left(1+n,-2\varphi \right)={\int }_{-2\varphi }^{\infty }{t}^{n}{e}^{-t}dt$ is the incomplete gamma function defined as $\Gamma \left(a,x\right)={\int }_{x}^{\infty }{t}^{a-1}{e}^{-t}dt$, ^48^ the geodesic Eq. (34) becomes

43 $$\frac{{d}^{2}\varphi }{d{\sigma }^{2}}+\frac{2}{\sigma }\frac{d\varphi }{d\sigma }+{\varphi }^{n}=0.$$

Based on the Eq. (37), the Eq. (43) can be rewritten as

44 $$\frac{1}{{\sigma }^{2}}\frac{d}{d\sigma }\left({\sigma }^{2}\frac{d\varphi }{d\sigma }\right)+{\varphi }^{n}=0.$$

The Eq. (44) has the same formula as the Lane–Emden equation in astrophysics^45,49^. In astrophysics, the pressure and density variations are estimated for self-gravitating spheres of plasma, such as stars. The Lane–Emden equation reported by astrophysicists Jonathan Homer Lane and Robert Emden is used to calculate the hydrostatic equilibrium based on the potential gradient, the density, and the pressure gradient. Since Poisson's equation connects the potential with density, Poisson's equation for the gravitational potential of a spherical polytropic fluid is given by the Lane–Emden equation as Eq. (44). $n$ is the polytropic index, defined as $P=K{\rho }^{1+\frac{1}{n}}$, in which $P$ is pressure, $\rho$ is density and $K$ is a constant. As $\mathrm{n}$ goes to infinity, the Lane-Emden equation reduces to the Emden-Chandrasekar equation. This result shows that the relationship between the density and radius also follows the LAP. Remarkably, the statistics-related equations all have the same type of variable reflection angle.

### The solutions of three drag force equations in fluid physics

Moreover, three equations in fluid physics can also be described as geodesic Eq. (16) under a specific given reflecting angle and potential. In the first case, given the reflecting angle

45 $$R=-6\pi \eta r/m,$$

46 $$U\left( {\varphi ,\sigma } \right) = C_{0} e^{ - \varphi + \sigma } \left( { - \varphi + \sigma } \right)\quad {\text{or}}\quad U\left( {\varphi ,\sigma } \right) = 0,$$

and potential function geodesic Eq. (16) becomes

47 $$m\frac{{d}^{2}\varphi }{d{\sigma }^{2}}=6\pi \eta r\frac{d\varphi }{d\sigma }.$$

The Eq. (47) has the same formula as that of a drag force equation in Stokes's law: $\frac{{d}^{2}x}{d{t}^{2}}=6\pi \eta rv=6\pi \eta r\frac{dx}{dt}$. In Stokes's law, the drag force exerted on a spherical object in a viscous fluid can be described as $F=-6\pi \eta rv$, where $\eta$ is the dynamic viscosity, $r$ is the radius of the spherical object, and $v$ is the flow velocity relative to the object^50^. Notably, the particles moving through a fluid at relatively slow speeds (with no turbulence) follow the LAP in displacement-time space.

In the second case, given a different reflecting angle (constant) of

48 $$R=-\frac{\rho {C}_{d}A}{2m},$$

and a potential function $U(\varphi ,\sigma )$ of

49 $$U\left( {\varphi ,\sigma } \right) = C_{0} e^{ - \varphi + \sigma } \left( { - \varphi + \sigma } \right)\quad {\text{or}}\quad U\left( {\varphi ,\sigma } \right) = 0$$

The Eq. (16) becomes

50 $$m\frac{{d}^{2}\varphi }{d{\sigma }^{2}}=\frac{1}{2}\rho {C}_{d}A{\left(\frac{d\varphi }{d\sigma }\right)}^{2}$$

The Eq. (50) has the same formula as the drag equation, $F=\frac{1}{2}\rho {C}_{d}A{v}^{2}$, where $\rho$ is the density of the fluid, $v$ is the speed of the object relative to the fluid, $A$ is the cross-sectional area, and ${C}_{d}$ is the drag coefficient^51^. The drag equation is used to calculate the force of drag experienced by an object due to movement through a fully enclosing fluid, such as lift-induced drag. The drag equation is relevant to wings or a lifting body, in which wave drag occurs when a solid object moves through a fluid near the speed of sound or along a fluid boundary. Interestingly, the trajectory of a particle high-speed moving in displacement-time space follows the LAP.

In the third case, given a different reflecting angle (constant) of

51 $$R=c/m,$$

and a potential function $U(\varphi ,\sigma )$ of

52 $$U\left(\varphi ,\sigma \right)={e}^{\varphi +\sigma }\left[-\frac{k}{4m}+\frac{k}{2m}\varphi \right],$$

The Eq. (16) becomes

53 $$m\frac{{d}^{2}\varphi }{d{\sigma }^{2}}+c\frac{d\varphi }{d\sigma }+k\varphi =0.$$

The Eq. (53) has the same form as the equation of 1-D damped vibration. The external forces include two terms with displacement and velocity. For 1-D damped vibration with an object under an elastic force ${F}_{e}=-kx$ and a resistance ${F}_{r}=-\mathrm{c}v$ (where $k$ is the elastic coefficient and $c$ is the viscous damping coefficient), the displacement and time satisfy the differential equation $m\frac{{d}^{2}x}{d{t}^{2}}=F={F}_{e}+{F}_{r}=-kx-cv$, i.e., $m\frac{{d}^{2}x}{d{t}^{2}}+c\frac{dx}{dt}+kx=0$. The result suggests that the trajectory of this particle follows the LAP in displacement and time space.

## Discussion

**Figure 2:**
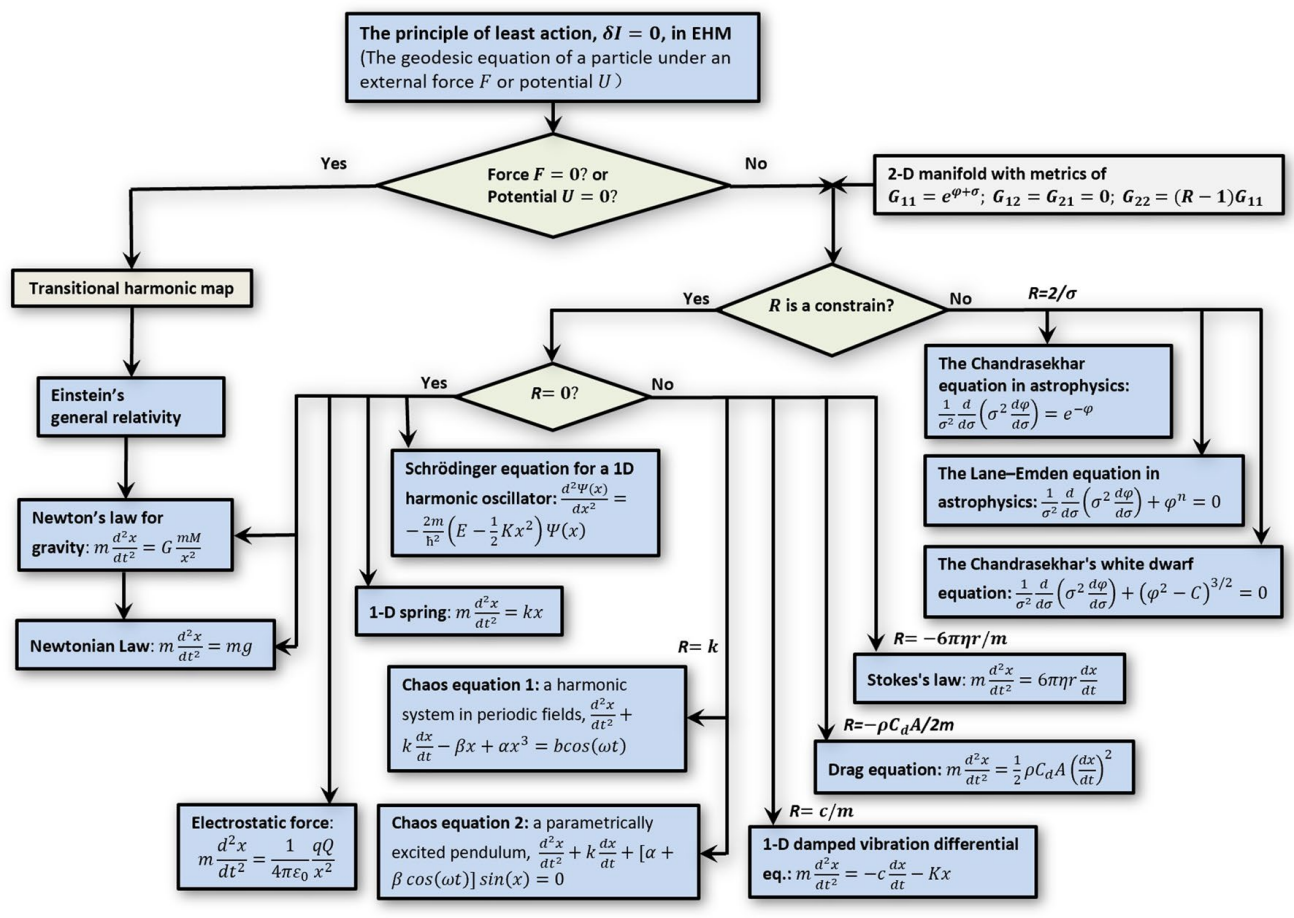
The family tree of the physics equations connected by the EHM and LAP. The physics equations can be derived from an universal geodesic equation (defined by LAP) in the EHM under a set of special given potentials, U, and reflecting/projecting angles, R. Those physics equations can be sorted and classified with respect to the reflecting angle and displayed as a family tree. Considering that the EHM contains the traditional HM under a vanished potential, the geodesic equation certainly containing the solution of Einstein’s general relativity is also connected to the geodesic equations that contain the solutions of the Schrodinger equations, classical equations of chaos, fluid physics and statistics-related equations in astrophysics. Our hypothesis is that the LAP on the Finsler manifold (used in defining the geodesic equation in EHM) is the universal rule of the universe.

“God does not play dice with the universe” is one of Albert Einstein's most famous quotes. Einstein and many scientists believe the trajectory of any object/particle should be smooth and differentiable, instead of having sharp edges, discrete chunks, or granular space-time. In this paper, we showed that the geodesic equation (the Eq. (11)) of EHM connects many physics equations. Although those popular equations have significantly different formulas, they are all solutions of an universal geodesic equation in EHM. The relationships among those equations can be sorted and classified based on their potentials and projection angles. The connections could be used to reveal a family tree of physics equations (Fig. 2) for a new perspective to understand our universe.

The potential term in the EHM can be understood as a super-force (or universal force) on a Riemannian surface. The super-force changes the local coordinate into a nonlinear form (Fig. 1). The geodesic equation with a super-force on the Riemannian surface can be understood as a standard geodesic equation on a Finsler surface. Thus, we believe the ultimate rule of the universe should be the LAP on a Finsler surface.

**Figure 3:**
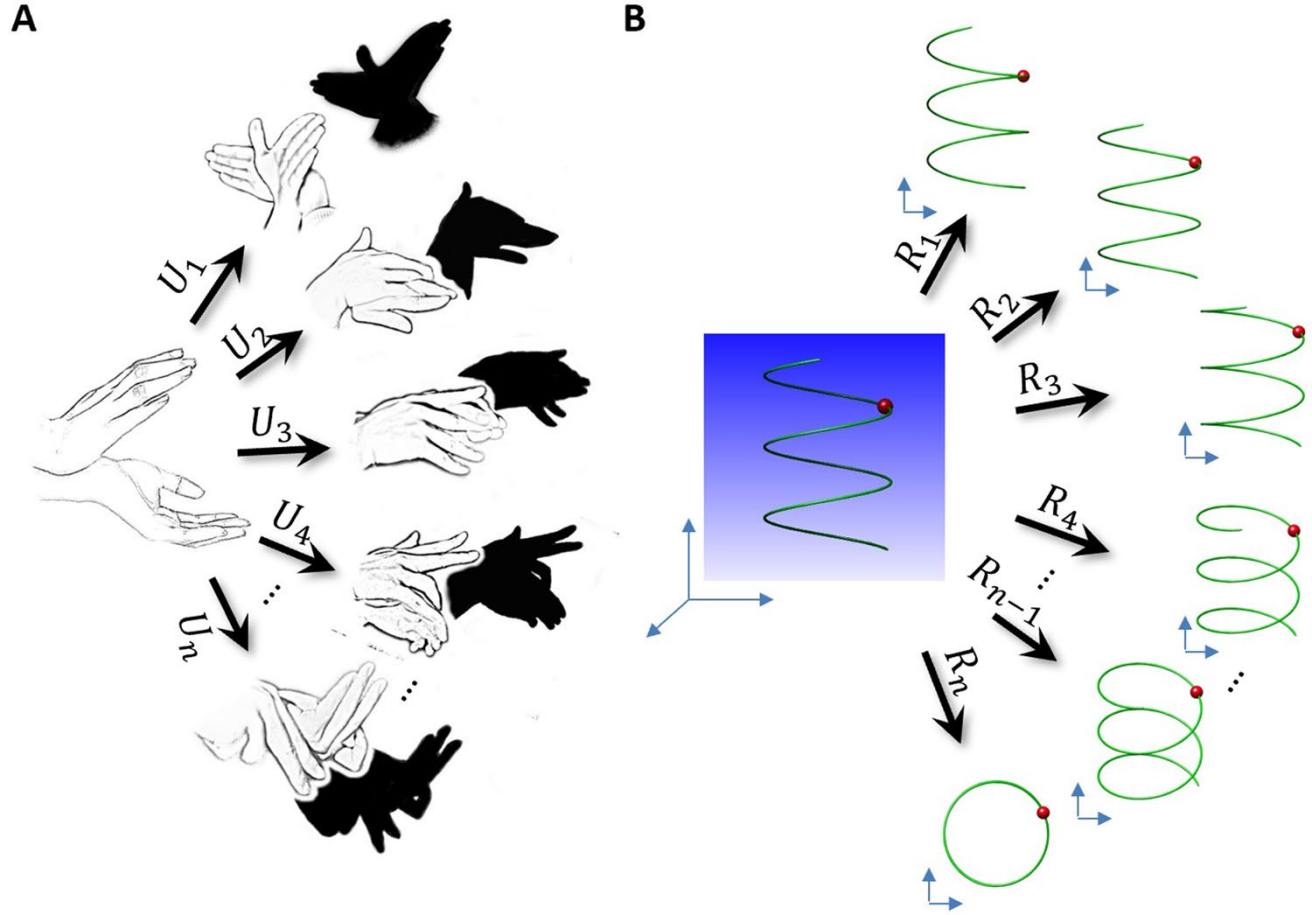
Schematics of the shadowgraphs showing the influence of a 3-D object on its 2-D projection.* (A)* When the fingers are twisted into a complex shape under a “weird” driving force (or potential, *U*_*1*_*, U*_*2*_* … U*_*n*_*.*), the “weird” 3-D finger shapes can be projected into simple and easily recognized animal shapes, suggesting that the complex formula of the potentials is due to the limitations of the description capability of low-dimensional space. **(B)** Moreover, a simple projecting/reflecting angle can also be used to generate a very complex projection. As an example, a simple trajectory of a particle moving along a spring could be projected into a series of complex trajectory shapes via different projection angles, such as *R*_*1*_*, R*_*2*_*, …, R*_*n*_. Some projections contains non-differitiable harp edges, while some positions are sharing with two or more fragments of the projection trajectories.

Although the formulas of the potentials shown in this paper are complex and unusual in form, it is because we are describing the super-force of Reimann space from our low-dimensional observation perspective. Using the formulas of our visualized low-dimensional universe to describe a high-dimensional super-force on a Riemannian surface is limited by the capability of description. To understand this statement, we can borrow the examples of “shadowgraphs” in the art of performing, as shown in Fig. 3A. The shapes of the fingers of the human hand (as the LAP or the universal geodesic equation) can be twisted into many complex and “weird” shapes under different forces (similar to our given potentials). However, the complex and weird shapes are hard to discripted, but their projections are simple  and with well-recognized shapes, such as the animals’ shadows (Fig. 3A). In this case, to discript how the 3-D figures were twisted from the discription of 2-D are rather complex.

Similarly, the reflecting/projecting angle, R, defining the metrics in the Eq. (16) also appears to be important in generating the physics equations. The simple formula of the reflecting/projecting angle rules the creation of complex equations, such as that for the chaos equations. To understand how a simple reflecting/projecting angle can have such power to create complex formations, we here  borrow another shadowgraph, as shown in Fig. 3B. For a simple trajectory of a particle that travels along a 3-D “spring”, this spring shaped trajectory can be projected into a series of complex trajectories in 2-D (Fig. 3B). Trajectories (other than a circle) are usually difficult to be described by equations, which means the complexity of the equations in our observation world could be a simple equation in a higher dimensions. The complexity may be generated by the limitations of the formulas that used in our living world.

Notably, the projection angle does not have to be a constant but could be a function of the coordinate (such as the three equations in astrophysics). The changing of reflecting/projecting angle can be understanding as the motion of viewing angle, which similar to our observation of the moving moon while driving, or the motion of a tree shadow under the sun induced by the spin of the earth. There is no force driving the motion of the tree shadow; the movement occurs due to the projection angle changing. Therefore, some physics phenomena observed in our living world may be caused by the motion of our living world against other universe. The change in projection angles may reflect the relative movement between the universes.

One hypothesis to understand quantized and discrete phenomena described in quantum physics in term of a smooth and differentiable trajectory shown in the geodesic equation in EHM is that, the object’s smooth and differentiable trajectory was conducted in a high-dimensional space, and its trajectoru occationally penetrates our measurable living world, left a series of isolated observation points. We discripted the isolated points as quantized and discrete trajectories in quantum mechanics. When the trajectory crossed a same observation point for several times, we can not measure where it was from and where it was going to. In this case we may introduce the the statiscs or probility to discript the observation. A simple understanding of above description could be that, when a needle penetrates a folded paper, an ant or bacteria on the paper could observe only a series of isolated holes and may describe the motion of the needle as a series of quantized and discrete trajectories, since they do not know their living space is falt or folded. Similarly, we may also live on the surface of a low-dimensional folded world, and the distances we observe as discrete chunks may be nearby and form a smooth and differentiable trajectory in a high-dimensional world. Based on this hypothesis, the electron shells within an atom may be folded together in a high-dimensional world; as a result, the electron travel time between shells is extremely short.

The connections among the physics equations shown in the EHM may lead to a new thinking way about our universe. The validation of the EHM requires substantial experimental evidence. We are opening the door for opportunities to discuss and collaborate with experts to design experiments to test the EHM theory. The experiments could be aimed at understanding the relationship between chaos and quantum enchantments^52^ and/or the experimental observation of superfluid and superconducting on atomic-molecular vortices by Raman spectrum^53^.

## Conclusion

In summary, the EHM theory provides an orthogonal view to superstring theory in understanding the ultimate rule of our universe. The ultimate rule can be described as LAP on a Finsler manifold. The connection among the physics equations provides a pathway to find the intrinsic connection between the ultimate universe and our observed living world via the family tree of physics equations (Fig. 2).
